# Moving the Needle: A 50-State and District of Columbia Landscape Review of Laws Regarding Pharmacy Technician Vaccine Administration

**DOI:** 10.3390/pharmacy7040168

**Published:** 2019-12-10

**Authors:** Deeb Eid, Joseph Osborne, Brian Borowicz

**Affiliations:** Department of Pharmacy Practice, Ferris State University, Grand Rapids, MI 49503, USA; osborj14@ferris.edu (J.O.); borowib@ferris.edu (B.B.)

**Keywords:** pharmacy technician, vaccination, delegation, regulations, statutes, board of pharmacy, practice of pharmacy

## Abstract

Pharmacy technicians are essential for inner workings of pharmacy teams and their depth of involvement in roles continues to evolve. An innovative role for pharmacy technicians, administration of vaccines, has emerged. With Idaho, Rhode Island, and Utah recently implementing changes that allow pharmacy technicians to safely perform this role, the need arose for a detailed examination of the law climate in all 50 states and the District of Columbia. A nine-question survey was sent out to all 51 state boards of pharmacy inquiring to legislative and regulatory environment of pharmacy technician vaccine administration. Additionally, a protocol driven, peer-reviewed process of state-specific regulations and statutes revealed categorized trends pertaining to this topic. Each state was classified per protocol into four different categories. The categorization resulted in identification of nine states in which pharmacy technician administered vaccination may be considered “Not Expressly Prohibited”. A majority of states were categorized as prohibited (either directly or indirectly). Board of pharmacy respondents (43%) reported varying viewpoints on technician administered vaccines. While three states (Idaho, Rhode Island, Utah) have already made changes to allow for pharmacy technician administered vaccinations, opportunities exist for other states to consider changes to statutes or rules.

## 1. Introduction

Vaccines remain one of the most cost-effective preventative health measures available and have been estimated to reduce direct financial burden on healthcare by $9.9 billion [1]. Additionally, pharmacies represent the second most common location for an adult to receive an influenza vaccination [2]. With an estimated 42,000 adult and 300 child deaths per year on average attributable to influenza alone, opportunity exists to improve access to this crucial preventative health intervention through expansion of patient access to vaccinations [1]. The World Health Organization (WHO) estimated that vaccinations prevent between two and three million deaths each year [3]. Kamal et al. identified various factors that may contribute to low rates of vaccination such as apathy, misconceptions, cost, distance to clinics, wait times, and inconvenient hours [4]. When it comes to barriers to receiving immunizations, less talked about or mentioned are the statutes and regulations surrounding them or who may be authorized to provide them. Given that pharmacies are one of the most accessible health destinations for the general public, they have served as a gateway to increase vaccination rates and improve access to care. According to data reported by the American Pharmacist Association (APhA) and National Alliance of State Pharmacy Associations (NASPA), pharmacists are authorized legally to administer vaccines in all 50 states and D.C. [5]. Pending a couple of states that have worked on recent law changes (New Jersey, New York), student pharmacists (interns) will soon be able to administer vaccines in all states as well [5,6,7].

As pharmacist roles continue to evolve over time, so will those of pharmacy technicians. Pharmacists’ professional delegation has become a key shift towards ensuring workload allocations and safe practices can remain intact. Working together with pharmacists and student pharmacists, pharmacy technicians play a critical role in impacting public health. Technicians represent a key opportunity to add a team member to help attribute to the public health initiative of increasing access to vaccinations. More recently, a technical but seemingly innovative role for pharmacy technicians, administration of vaccines, has emerged. With recent outbreaks of vaccine preventable diseases, and patient safety at the forefront of missions of boards of pharmacy, the public may benefit from adding another pharmacy team member to help increase access to vaccinations.

In 2016–17, Idaho actively underwent a rule rewrite which included adding language that directly permitted pharmacy technicians to administer immunizations [8]. With this, they became the first state within the U.S. to do so and also became the first state to actively involve pharmacy technicians in a training program and administration at local pharmacies. More recently in 2018, Idaho broadened language to allow for delegation utilizing professional judgement and therefore laws became “silent” on the topic. Seeing that statutes and regulations are silent, this could be interpreted as technically not an illegal task to delegate and perform, or in legal terminology; permissive. This also raises an interesting concept of silence within law and how state agencies or others may interpret these findings. Rhode Island became the second in late 2018 to promulgate rules and Utah made changes to their statewide vaccine protocol shortly after [9,10].

To date, three states have made changes within scope of practice to include pharmacy technician administration of vaccinations, including Idaho, Rhode Island, and Utah with others pending [8,9,10,11]. What may not be as apparent are the statutes and regulations surrounding allowance, prohibition, or silence in all 50 states and D.C. The purpose of this survey was to review and compile data surrounding the statutes and regulations pertaining to pharmacy technician administration of immunizations. With the data analyzed, the goal of the project was to provide a national overview of state specific language, citations, and examples of law variations. This report may serve as an informative reference for discussion or changes that could be made to either statutes or regulations per respective states. 

## 2. Materials and Methods 

Data collection consisted of a two-pronged approach in which a state-specific board of pharmacy survey and a peer-reviewed classification process were conducted. This project was found to be exempt from Institution Review Board (IRB) approval by the Ferris State University IRB (IRB-FY18-19-58).

In October 2018, a nine-question survey constructed in QuestionPro was emailed to 50 state boards of pharmacy, including the District of Columbia. Contact information for each board of pharmacy had been identified using a publicly available complied contact list collected via the website www.stateside.com. Any failure to deliver notices or kickback messages required direct communication with that specific board for updated contact information. Instructions for completion of the survey along with consent were included in an introduction with a given time estimate of 5–15 minutes. Those contacted were informed that participation in the survey was voluntary, and that all responses, including non-response, would be recorded and published. No personal or demographic information was collected, and all respondents remained anonymous. Representatives were asked to indicate the state agency their response represented. See Table A1 in Appendix A, for a comprehensive list of survey questions. 

The survey was disseminated 24th October 2018, with a two-week timeline of 6th November 2018. Reminders to complete the survey were sent 29th October 2018, one week before the deadline via email to the same contact address used initially. Data was assimilated from QuestionPro into a shared data collection program for evaluation. The classification process contained two-steps: 1) manual review of state-specific statutes and regulations, and, 2) group peer review of manual review results. The peer-review classification process began in November 2018 and concluded in May 2019. 

**Figure 1:**
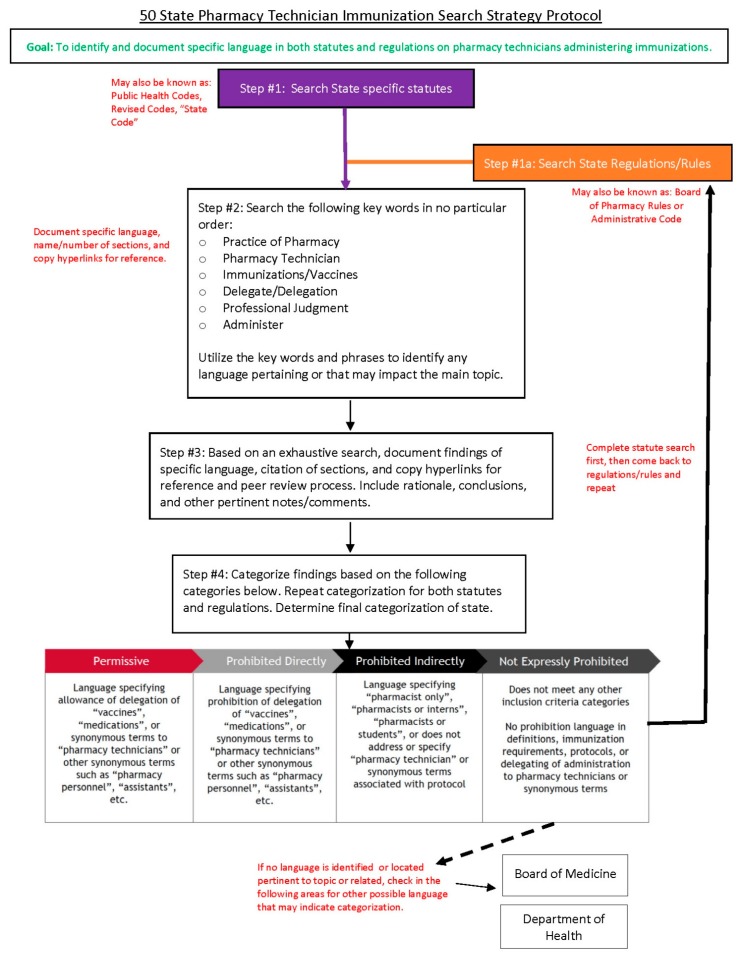
Manual Review Search Protocol.

While the manual survey categorization methods could be considered somewhat unique, a review of methodology from Tzanetakos et al. and Stewart et al. helped build the foundation [12,13]. The manual review of state-specific statute began with the division of 50 states (and the District of Columbia) alphabetically (Alabama through Missouri; Montana through Washington D.C.) into two sets consisting of 25 and 26 states. One author was then selected to review each of these divisions, with selection of author review of these groups arbitrarily chosen. The protocol in Figure 1 was used as a standardized approach to research, identify, and document state-specific results: State-specific statutes (Public Health Code, Revised Code, and “State Code”) were reviewed through the use of the following key words: “Practice of pharmacy”, “Pharmacy Technician”, “Immunizations [or] Vaccines”, “Delegate [or] Delegation”, “Professional Judgement”, and “Administration”. If language was found in state-specific statute after the search using these keywords, it was documented into one of four categories discussed in the Results section (Section 3). If no language was found in state-specific statute, that same search protocol was directed at the matching state’s Board of Health and Board of Medicine. At this step, regardless of if language had been identified or not, it would be categorized per protocol and documented into the shared data collection program into one of the following categories: Not Expressly Prohibited, Prohibited Directly, Prohibited Indirectly, and Permissive. This process was then repeated for that identical state’s regulations (Board of Pharmacy Rules or Administration Code) with categorization and documentation occurring in an equal fashion. After both statutes and regulations had been documented for a state, this entire procedure was repeated until all states had been categorized. Upon completion of data collection, the author assigned to one set subsequently peer reviewed the opposite set via the same protocol, ensuring that every data point had received equal analysis. A color-coded system (red, yellow, green) was used to compare agreement in findings (complete disagreement, agreement with discourse, and complete agreement) to facilitate the group peer-review process. 

The group peer-review process began shortly after the conclusion of the manual review. A final step of the peer review process involved having the primary investigator review all entries to confirm categorization or settle differences identified. All authors met and discussed results of data collection using citations documented in the shared data collection program. Discussion occurred until every data point had been finalized to facilitate bias mitigation and settle any discrepancies. This peer review methodology known as triangulation was adopted from Farmer et al. [14]. The primary investigator also reconciled survey data as another comparator. Final data categorization was then recorded. 

Definitions of Permissive, Prohibited Directly, Prohibited Indirectly, and Not Expressly Prohibited are encapsulated in Table A2 in Appendix A.

## 3. Results

### 3.1. Board of Pharmacy Survey Results 

Of the 50 states and the District of Columbia polled, 22 (43%) states successfully completed the survey. State boards of pharmacy who finished the survey included the following: Arizona, Hawaii, Idaho, Iowa, Kansas, Kentucky, Louisiana, Maryland, Massachusetts, Minnesota, Nevada, New Hampshire, North Carolina, North Dakota, Ohio, Oregon, Rhode Island, Texas, Vermont, Virginia, Washington and Washington D.C. One state submitted past the 6th November deadline (submitted 9th November). Of the states responding, 16 (72%) reported that there were statutes (state legistlation, public health code…etc.) that prohibit pharmacists from delegating the task of vaccine administration to a properly trained pharmacy technician. For the similar question regarding regulation, 13 (59%) states reported prohibition via rule. Eight (36%) respondent states answered “yes” to if there had been any discussion from their board on this topic to date. Table A3 in Appendix A outlines a few selected free responses from the survey. For both question eight and question nine of the survey, six (27%) of the responding states gave an answer equivalent to “no comment”. When asked about initial impressions in question eight, 17 (77%) free responses were recorded. In question nine, when asked about risks, 15 free responses were recorded (68%). All 22 states (100%) provided statute or regulation citation when required. Overall, there were also multiple free responses that respondents declined to answer or did not directly answer the question(s). For those purposes, Table A3 includes free responses that were thought provoking and/or provided insight based on the question asked. 

### 3.2. Peer-Review Classification Results 

**Figure 2:**
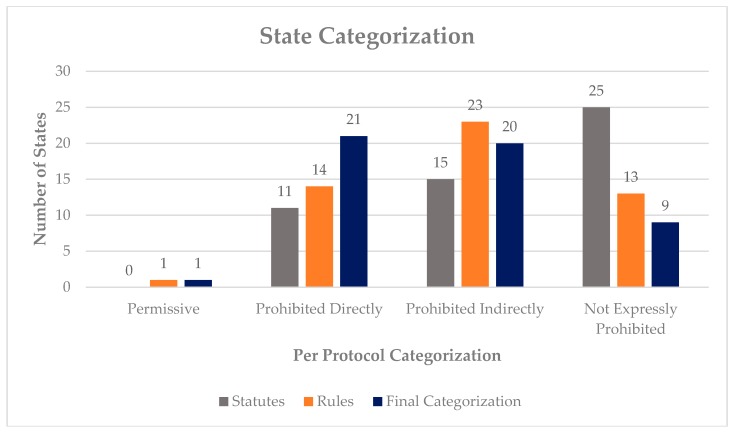
State Categorization (including D.C.).

The following data was collected per protocol from all 50 states and D.C.: overall, one (2%) state was found to be Permissive, 21 (41%) states were classified as Prohibited Directly, 20 (39%) states were classified as Prohibited Indirectly, and nine (17%) states were classified as Not Expressly Prohibited. The above classification considered both statute and regulation and the stricter of the two findings per state (including D.C.). Regarding statute only, zero (0%) states were found to be Permissive, 11 (21%) states were classified as Prohibited Directly, 15 (29%) were classified as Prohibited Indirectly, and 25 (49%) of states were classified as Not Expressly Prohibited. When regulations were examined, 1 (2%) state was classified as Permissive, 14 (27%) states were found to be Prohibited Directly, 23 (45%) states were classified as Prohibited Indirectly, and 13 (25%) were classified as Not Expressly Prohibited. See Figure 2 for a graphical representation of the data. To further provide examples of how the categorization occurred, selected examples that were most transparent are provided below.

Rhode Island, being the only state to expressly permit pharmacy technicians to administer immunizations within rules, is the example of a Permissive state. Statute contains no explicit prohibitions when examining the definitions of “pharmacist” or “pharmacy technician” and includes the following definition of “practice of pharmacy” found in RI Gen L 5-19.1-2(x): “Practice of pharmacy...includes...administration of adult immunizations in accordance with regulations and training requirements promulgated by the department of health” [15]. Considering regulation, administrative code 216-RICR-40-15-1.11 (8, b.) outlines “A technician II who has completed a recognized certificate training course on appropriate immunization administration technique and holds a current basic cardiopulmonary resuscitation (CPR) training certificate, shall be permitted to administer vaccinations under the direct supervision and with the authorization of an immunizing pharmacist…” [16]. Rhode Island’s responses to the survey also indicated answers of “no” to questions two and four. The above language is an example of Permissive categorization.

South Carolina provides example of a state in which pharmacy technicians are Prohibited Directly. Within statutes, Section 40-43-190 (B,3) clearly prohibits with the following language: “A pharmacist may not delegate the administration of vaccines to a pharmacy technician or certified pharmacy technician” [17]. Seeing that a majority of pertinent language relating to pharmacy technicians or vaccines are within statute, there were no prohibitions found within regulations, therefore deeming Not Expressly Prohibited. Interestingly, prohibition is further reinforced through the Pharmacy Policies and Procedures document within Approved Technician Duties Policy and Procedure #140 which states “The pharmacy technician is prohibited from performing the following functions:...administering immunizations” [18]. Although no clear indication, authors assumed Policy #140 is referring back to Section 40-43-190 (B,3), as the document often cited other specific statutes. South Carolina additionally prohibits technician immunization through language within statewide protocol: “A pharmacist may not delegate the administration of vaccines to a pharmacy technician…” [19]. According to survey results, South Carolina started a response but failed to complete the survey, therefore there was no data to reconcile during the peer review process. As documented in South Carolina law, this state was categorized as Prohibited Directly.

North Carolina illustrates the Prohibited Indirectly category. NC Gen Stat § 90-85.3.i1 outlines that “‘Immunizing pharmacist’ means a licensed pharmacist who meets all of the following qualifications.[lists qualifications]” [20]. This statute does not implicitly disallow pharmacy technicians, yet it does specifically list a pharmacist. Regulations in North Carolina also provided Prohibited Indirectly language as seen in 21 NCAC 46.2507: Administration of Vaccines by Pharmacists with “A) an Immunizing Pharmacist or a Pharmacy Intern who is under the direct, in-person supervision of an Immunizing Pharmacist;” [21]. It is worth noting that while pharmacy technicians may be technically prohibited via exclusion, a regulatory artifact exists that permits “(B) the patient at the direction of either an Immunizing Pharmacist or a health care provider” to administer their own immunization [21]. This indicates that a pharmacist or other healthcare professional may teach a layperson to administer their own vaccine, yet a trained pharmacy technician may not qualify. Because of the lack of specific prohibition of pharmacy technician immunization administration combined with the explicit listing of those who can administer, North Carolina was classified as Prohibited Indirectly.

Idaho was found to be an example of Not Expressly Prohibited categorization, being that pharmacy technician immunization was not defined within their statute or regulation per the protocol defined. In both statutes ID Code 54-1704 and regulations IDAPA Rule 27.01.01.100 no mention was made to pharmacy technicians being able, or unable, to provide immunization administration [22,23,24]. Rather within 27.01.01.100, it states “To evaluate whether a specific act is within the scope of pharmacy practice in or into Idaho, or whether an act can be delegated to other individuals under their supervision, a licensee or registrant of the Board must independently determine whether:…” and then lists a few lines of guidance. According to survey results, Idaho also answered questions two and four with “no”, indicating similarities with peer review findings.

Only one state was found to be categorized as Permissive (Rhode Island), which both the survey and peer-reviewed classification agreed upon. A total of 41 states (80%) were classified as Prohibited Directly or Prohibited Indirectly through statute or regulation. This finding was not surprising considering the minority of states (three) in which pharmacy technicians currently can administer immunizations [8,9,10,11]. The remaining two states (Idaho, Utah) which currently have pharmacy technician administration of immunizations are classified as Not Expressly Prohibited. This peer-review classification was in agreement with the results from the Idaho Board of Pharmacy survey. Utah board survey data was unavailable.

### 3.3. Comparison of Survey to Peer-Review 

When comparing survey respondent states to their collected peer-review data, the authors were in agreement with the state board of pharmacy 16 out of 22 times (73%) regarding statutes. When comparing data for regulation, the authors were in agreement with survey respondents 16 out of 22 times (73%). Of the disagreements, the authors disagreed with the categorization of both statute and regulation with four states of the 22 states who completed the survey. Four state board survey findings (Kentucky, Louisiana, Minnesota, and Washington) were misaligned with the results from the peer-reviewed classification. Of note, three of these states (Kentucky, Minnesota, and Washington) reported board survey information that was more conservative (i.e., the authors found their state to be Not Expressly Prohibited rather than Prohibited Indirectly) than the peer-review classification, while Louisiana reported a more liberal interpretation (i.e., the authors found their state to be Prohibited Indirectly rather than Not Expressly Prohibited) than the peer-review classification. An encompassing state-specific compilation of both survey results and peer-reviewed classifications complete with rationale is available in Table A4 in Appendix A.

## 4. Discussion

The vision for the project was to be eventually used as a tool for all interested parties. Stakeholders may consider findings, elicit discussion, and spread awareness across the nation in the future. State agencies or other stakeholders may enter discussions on the topic and want to better understand the landscape of laws from a national overview. This project does not serve as legal interpretation or was not meant to be misinformed or misconstrued as so.

As mentioned prior, three states, Idaho, Rhode Island, and Utah have expanded practice by making regulatory or protocol changes. From the federal level, the Commissioned Corps of the U.S. Public Health Service announced that credentialed pharmacists have the chance to provide federal pharmacy technicians an opportunity to obtain training to administer vaccines [24]. In Whiteriver, Arizona within the Indian Hospital, pharmacy technicians have administered vaccines to patients of all ages (including children) with oversight from a federal pharmacist [25]. With change on the horizon and precedent set, investigation and categorization of laws in other states were identified as gap areas within published literature.

The topic itself does not lend to an array of literature examples or studies to draw from, therefore this presents as a novel area to provide insight. A comparator study would be the 2015 study by Stewart and colleagues which examined the state laws and standing orders for immunization services. Within this study, authors did not examine pharmacy technicians specifically, but looked more broadly at non-physician health professionals. Interestingly, it was found that medical assistants (comparable to pharmacy technicians in training, education, and roles) had delegated authority to administer vaccines in fourteen (14 ) states, own authority in one (1) state and laws were silent within thirty-six (36) states and D.C. [13]. State laws also varied, but a general trend noted was that physicians are able to delegate the task of vaccine administration to medical assistants in many states. The study by Stewart and colleagues had conceptual similarities but did not endure a triangulation peer review methodology. Of note, training and education requirements of medical assistants and pharmacy technicians also vary from state to state, which can make it challenging to argue that education requirements are mandatory for one to succeed outside of a training program designed specifically for the task. 

When considering the topic of pharmacy technician administered vaccines within law, there arose a few theme areas investigators identified for state agencies and others to consider after completion of data analysis and discussing results. One area includes the training requirements and availability of a training program. McKeirnan et al. and Washington State University (WSU) developed a training program that is specific for pharmacy technicians [26]. The program was designed to be less time intensive or in-depth (2-hour self-study, 4 hour live) compared to the pharmacist/student program (~20 hours) with a clear separation of the technical versus clinical aspects of vaccine administration [27]. There have also been speculations as to if the WSU program has been recently acquired by a national association and may soon be featured as a nationally recognized program.

The second theme to consider includes the platform by which changes would need to occur. Would the state require rule promulgation or amendments, a statute change, or both? Are amendments to statewide protocols or collaborative practice agreements needed? Maybe a state currently has no true prohibitions and it may be up to employers to kickstart the practice model? While there were (9) states identified in the categorization of Not Expressly Prohibited, readily available opportunities may exist to begin implementation of technician vaccine administration within these states. It serves important for stakeholders to work closely with state agencies, boards, and others on moving initiatives forward. If rules or statutes need to be amended or changed, states could also consider utilizing a pharmacist delegatory model. This model would allow for pharmacists to use professional judgement to delegate technical tasks such as immunization administration to support personnel. Similar to physician, optometry, or dentistry models, it may enable pharmacists to practice and manage their practices at a level that may be conducive to the public and patient safety. This model also supports the recent NABP Task Force developed to investigate moving pharmacy to a “Standard of Care” model [28]. To continue to evolve, the profession of pharmacy must evolve as a team and utilize teamwork to provide patient care that is safe and effective.

A third and final theme to consider involves fears and emotions that arise when considering any type of changes. Atkinson et al. describes in depth the typical fears and emotions brought up whenever having discussions on the topic as deliberated in initial discussions in Idaho [29]. Many points of concern highlighted within Atkinson et al. are theories based on precautionary principle, and lack evidence to support rationale. To properly evaluate the topic, it is crucial to consider what concerns are present, but to not let theories supersede and prohibit positive public health initiatives backed by evidence. Within the survey, when asked about safety concerns or risks, comments trended towards being majorly positive on the topic. Boards mentioned key phrases such as “with the same training”, “if properly trained” or “just as untrained lay persons have”, indicating a sense that proper training is key. A minority of respondents mentioned phrases such as “clinical education”, “clinical judgement”, or “the medical community may not be accepting”. Therein lies the differentiation of clinical versus technical knowledge and roles. McKeirnan et al. demonstrated safety data which showcased 953 immunizations delivered by technicians with zero adverse events [26]. Three other studies, Burgess et al., Zahn et al., and Coleman et al. all demonstrated that even laypersons exhibited positive safety data when taught to administer their own vaccines [30,31,32]. Bertsch et al. surveyed pharmacists who supervise immunizing technicians and showed that opinions revealed positive morale of teams and can help to increase the number of vaccinations given by the pharmacy [33]. Not only has this practice shown safe data, but also has demonstrated another route to increasing public access to vaccines, a highly impactful public health initiative. Similar fears or emotions often arise when other expanded roles of pharmacy technicians are discussed. Within other well studied roles such as Technician Product Verification (Tech-Check-Tech), Verbals, Transfers, Clarifications, or, Point of Care Testing, evidence suggests similar affirmations around positive safety data and historical success in various jurisdictions for over 40 years for some roles [34,35,36,37,38,39].

Findings from the manual scanning of all states may have been subject to investigator expectations. Naturally, a majority of states were expected to include language that directly or indirectly may prohibit pharmacy technicians from administering immunizations. The survey responses helped to provide investigators with a comparator for the manual survey. Seeing that all states did not participate in the survey, this is an obvious limitation. Another limitation was the search protocol may not have encompassed all possible language included in regulations or statutes. While the protocol was designed to include as many relevant keywords or areas as possible, there was a chance that areas may have been missed. Free responses provide a snapshot of thoughts, discussions, and considerations by various boards across the country. Overall, respondents seemed to showcase the notion that the topic has been of interest or brought up, therefore validating that law changes or continued discussions may come in the near future. 

## 5. Conclusions

Overall, a majority of states (41) were found to include language that prohibits administration of immunizations by pharmacy technicians. Nine (9) states were found to be Not Expressly Prohibited by the peer-review triangulation process. Two (2) (Idaho, Utah) of these nine (9) states currently allow pharmacy technician immunization administration with others undergoing discussion. This is of paramount importance when considering the seven remaining Not Expressly Prohibited states: Kentucky, Michigan, Minnesota, Nebraska, New Mexico, Tennessee, and Washington. Proponents of pharmacy technician administration of immunizations may consider these key states to explore implementation with opportunities for expansion of practice. Given the legal judgement needed to navigate the proximity of Prohibited Indirectly and Not Expressly Prohibited, stakeholders of pharmacy technician administered immunizations may wish to closely examine the wording in both statute and regulation in these states. Boards of Pharmacy have mixed responses when asked about the topic and discussions seem to be growing in prevalence throughout the country.

## Appendix A

**Table A1:** State-Specific Board of Pharmacy Survey Questions.

Question	Response Field
1. What State Board of Pharmacy do you represent?	Free Response
2. In your state, are there statutes (state legislation, public health code…etc.) that prohibit pharmacists from delegating the technical task of vaccine administration to a properly trained pharmacy technician?	Yes (if answered logic guided to #3) No (skip to #4)
3. Please provide citation to the specific statute(s) (state legislation, public health code…etc.) that prohibit pharmacists from delegating the technical task of vaccine administration to a properly trained pharmacy technician.	Free Response
4. In your state, are there regulations (rules, BOP rules…etc.) that prohibit pharmacists from delegating the technical task of vaccine administration to a properly trained pharmacy technician?	Yes (if answered logic guided to #5) No (skip to #6)
5. Please provide citation to the specific regulation(s) (rules, BOP rules…etc.) that prohibit pharmacists from delegating the technical task of vaccine administration to a properly trained pharmacy technician.	Free Response
6. Have there been any discussions from your board on this topic to date?	Yes (if answered, logic guided to #7) No (skip to #8)
7. Please briefly describe discussions that have occurred from your board on this topic.	Free Response
8. What initial impressions do you have about pharmacy technicians administering vaccinations?	Free Response
9. Do you believe there are any risks that can occur from a pharmacy technician administering a vaccine relative to a student pharmacist? Please explain:	Free Response

**Table A2:** Categorization Guidance Protocol.

Permissive	Prohibited Directly	Prohibited Indirectly	Not Expressly Prohibited
Language specifying allowance of delegation of “vaccines”, “medications”, or synonymous terms to “pharmacy technicians” or other synonymous terms such as “pharmacy personnel”, “assistants”, etc.	Language specifying prohibition of delegation of “vaccines”, “medications”, or synonymous terms to “pharmacy technicians” or other synonymous terms such as “pharmacy personnel”, “assistants”, etc.	Language specifying “pharmacist only”, “pharmacists or interns”, “pharmacists or students”, or does not address or specify “pharmacy technician” or synonymous terms associated with protocol	Does not meet any other inclusion criteria categories No prohibition language in definitions, immunization requirements, protocols, or delegating of administration to pharmacy technicians or synonymous terms

**Table A3:** Thought Provoking Open Responses to Survey Questions.

Survey Question	Survey Response(s)	Valid Responses
**Please briefly describe discussions that have occurred from your board on this topic.**	A: The concept is interesting but we are trying to gauge the public health impact of such a move. (would it increase immunization rates) Although we have no issue with allowing properly trained technicians to give immunizations/injections, we need to have better participation from pharmacies on providing immunization services as well as other extended services that the Board has championed (CLIA waived tests, collaborative agreement, tech check tech, etc.) A: This has been discussed recently and the Board voted to pursue legislation that would permit a pharmacist with the ability to delegate the administration of a vaccine to properly trained pharmacy staff. While no law currently exists that specifically prohibits the delegation of the administration, all laws pertaining to vaccine administration are specific to pharmacists.	8/22 (36%)
**What initial impressions do you have about pharmacy technicians administering vaccinations?**	A: The administration of an immunization is a technical task that has been successfully performed by lay persons. There is no reason to deny patients access to this. A: We follow the law. A: Surprise/shock. Followed by a dose of reality: Physicians, NPs, and PA-Cs don’t typically perform the administrations of vaccines. That’s typically done by a CNA or a CMA. A: There is a lack of standardized training of all pharmacy technicians. Many have never completed a pharmacy technician training program, and were ’grandfathered’ in after the training requirement went into effect. If allowed by the Board, there would need to be standardized training and validation or certification, with ongoing continuing education for those technicians. A: Staff have had discussion on whether this might be allowed as a non-discretionary function or a specialized function under the current statutory and regulatory framework.	17/22 (77%) **
**Do you believe there are any risks that can occur from a pharmacy technician administering a vaccine relative to a student pharmacist? Please explain:**	A: We have never heard anyone say they think it would be unsafe for technicians to administer a vaccine; we have, however, heard various boards raise concerns about what it would do to pharmacist employment and how it could upset the medical profession. Neither of those reasons are appropriate decision points for boards of pharmacy to consider. Safety, and safety alone, should be the consideration, and studies have been published demonstrating trained technicians can safely and appropriately take on this task, just as untrained lay persons have.A: With the same training, no I do not see any additional inherent risks merely because they are a technician. A: We follow the law. A: Believe that a properly trained technician can perform the technical administration of a vaccine just as safely as any other properly trained individual. Possible risk within the pharmacy environment are potential missed opportunities for the pharmacist to dialogue with their patients during the administration process. A: No. Anyone is capable of giving a bad shot whether it is a pharmacist, student or technician. A: The risks would be the same for both technicians and students.	15/22 (68%) **

** Responses were omitted if answers to the question were not given or “N/A”, “no comment”, were provided.

**Table A4:** Comprehensive Peer Review Results.

State	Statutes	Regulations	Conclusion	Documented Regulations or Statutes	Survey Results
Alabama	NEP	PRI	PRI	1) ALB Code Title 34-23-130 Pharmacy Technicians 2) 680-X-2-.14 (1),(2) (a,b)-The Role Of Technicians In Pharmacies In Alabama.	N/A
**Alaska**	PRI	NEP	PRI	1) Title 8 Ch 80 Article 2. Sec 08.80.168 2) Alaska Stat. § 08.80.168a	N/A
**Arizona**	PRI	PRI	PRI	1) AAC R-4-23-411 C1: non-delegation allowed 2) 32-1901-defines "administer" 3) 32-1974 defines immunization and states "pharmacist" multiple times	AZ --> Statute, cited 32-1974 as Prohibited, but missed definition of "Administer" which includes "by the practitioner’s authorized agent". 4-23-411 cited in survey
**Arkansas**	PRD	PRI	PRD	1) A.C.A 17-92-101-16-xi-C-i 2) Regulation 3-Pharmacy Technicians: 03-00-0005 a,b	N/A
**California**	PRI	PRI	PRI	1) 4052.8. Initiation and Administration of Vaccines; Requirements 2) 1746.4 Pharmacists Initiating and Administering Vaccines.	N/A
**Colorado**	PRI	PRI	PRI	1) 12-42.5-102 (31)(b) 2) 12-42.5-102 (30) 3) 12-42.5-116 (5) 4) 3 CCR 719-1.19.01.10 5) 3 CCR 719-1.19.01.20	N/A
**Connecticut**	PRI	PRI	PRI	1) Chapter 400j. Sec. 20-633 2) 20-633-Administration of Vaccine By Pharmacist	N/A
**Delaware**	NEP	PRI	PRI	1) 24 Del.C. 2502 Definitions-(23)(h) 2) 24 Del.C. 2507 License required (b) 3) 14.0 DE Regs Administration of Injectable Medications, Biologicals and Adult Immunizations 4) 14.1 DE Regs, 14.2 DE Regs	N/A
**Florida**	PRI	PRI	PRI	1) Title XXXII, Chapter 465.014 Pharmacy technician 2) Title XXXII, Chapter 465.003 Definitions 3) Title XXXII, Chapter 465.189 Administration of vaccines and epinephrine auto injection 4) 64B16-27.420 Pharmacy Technician—Delegable and Non-Delegable Tasks.	N/A
**Georgia**	NEP	PRD	PRD	1) OCGA Title 26-4-82: Duties requiring professional judgment; responsibilities of licensed pharmacist 2) Title 26-4-4: Definition of “practice of pharmacy” 3) Title 26-4-5 (1),(10),(32): Definitions 4) Rule 360-34-.01 Definitions 5) Rule 360-34-.02 Qualifications for Physician to enter a protocol 6) Rule 360-34-.03 Qualifications for a Pharmacist to enter a protocol 7) 360-34-.05 (6) Requirements of the Vaccine Protocol Agreement	N/A
**Hawaii**	PRI	PRI	PRI	1) §461-1 (2) (E. Administering) Definitions. 2) §16-95-86: Scope of practice of a pharmacy technician	§461-9 Pharmacist in charge; pharmacy personnel HAR §16-95-2: §16-95-86 Scope of practice of a pharmacy technician.
**Idaho**	NEP	NEP	NEP	1) Idaho Code Title 54-1704: Practice of Pharmacy 2) IDAPA Rule 27.01.01.100: Practice of Pharmacy: General Approach	Answered “No” to both Statute and Rule questions
**Illinois**	NEP	PRI	PRI	1) 225 ILCS 85/3 (4,b)-Definitions 2) 225 ILCS 85/9 (a)-Licensure as registered pharmacy technician. 3) Title 68-1330.50 (a)(b): Vaccinations/Immunizations (Qualifications, Protocols, Policies, and Procedures)	N/A
**Indiana**	PRI	PRD	PRD	1) IC 25-26-13-31.2: Administration of immunizations; emergency immunizations; immunization data 2) IC 25-26-13-31.5: Immunizations by pharmacist interns and pharmacist students; rules 3) IC 25-26-19-8: Prohibited activities of a licensed pharmacy technician 4) 856 IAC 4-1-1: Pharmacist Vaccinations Administered Via Protocol Authority 5) 856 IAC 4-1-3 Delegation of protocol authority	N/A
**Iowa**	NEP	PRD	PRD	1) 155A.3 (1,12): Definitions 2) 155A.4 (c): Prohibition against unlicensed persons dispensing or distributing prescription drugs-exceptions. 3) 155A.44: Vaccine and immunization administration 4) 155A.46 (3-6): Statewide protocols 5) 155A.33: Delegation of technical functions 6) 657-39.10 (155A): Vaccine administration by pharmacists-physician-approved protocol. 7) 657-39.11(155A): Vaccine administration by pharmacists—statewide protocol. Others to consider: 8) 657-3.22(155A) Technical functions. 9) 657-3.21(155A) Delegation of functions.	Answered “No” to both Statute and Rule questions Also provided comments on pursuing legislation
**Kansas**	PRD	NEP	PRD	1) 65-1635a (c). Administration of vaccine; education and reporting requirements; delegation of authority prohibited; "pharmacist" defined. 2) 68-2-20 Pharmacist function in filling a prescription.	Statutes --> KSA65-1635a Rules --> Answered "No"
**Kentucky**	NEP	NEP	NEP	1) 315.010 (21,22): Definitions for chapter. 2) 315.020 (4): Only pharmacists to supervise manufacturing of pharmaceuticals or practice pharmacy-Exceptions 3) 315.205: Notification of immunization to minor’s primary care provider 4) 201 KAR 2:045. Technicians.	Answered KRS 315.010(22) for both
**Louisiana**	PRI	PRI	PRI	1) RS 37:1218: Administration of influenza immunization 2) RS 37:1218.1: Administration of immunizations and vaccines other than influenza immunizations 3) Title 46, Chapter 5: 521: Prescription Orders to Administer Medications 4) Title 46, Chapter 9: 907: Scope of Practice (Technicians)	Answered "No" to for both
**Maine**	PRD	PRI	PRD	1) Title 32, Chapter 117: 13834 Prohibited Acts 2) 13831: Authority 3) 02-392-4A: Administration of Drugs and Vaccines 4) 02-382-7: Licensure and Employment of Pharmacy Technicians	N/A
**Maryland**	PRD	PRD	PRD	1) 12-6B-06: Authorized and prohibited acts 2) Code of Maryland Regulations: 10.34.34.03 (7): Delegated Pharmacy Acts 3) 10.34.32.03: Requirements to Administer Vaccinations	Referenced 10.34.32.03 (specific to immunization education, requirements for pharmacists) for both questions
**Massachusetts**	NEP	PRD	PRD	1) Section 24B1/2: Pharmacist collaborative practice agreements; collaborative drug therapy management 2) 247 CMR 8.00 (.04-4e): Pharmacy Interns and Technicians	247 CMR 8.04 referenced, nothing for Statute
**Michigan**	NEP	NEP	NEP	1) MCL 333.17739: Pharmacy technician functions; licensure 2) MCL 333.16215: Delegation of acts, tasks, or functions to licensed or unlicensed individual; supervision; rules; immunity; third party reimbursement or worker’s compensation benefits. 3) R 338.3665 Performance of activities and functions; delegation. 4) R 338.486: “Medical institution” and “pharmacy services” defined; pharmacy services in medical institutions. 5) R 338.490 (5): Professional responsibility; "caregiver" defined.	N/A
**Minnesota**	NEP	NEP	NEP	1) 151.01 Subd.27 DEFINITIONS (Practice of pharmacy) 2) 151.102 Subd.1. Pharmacy Technician 3) 6800.3850 Subp.2 Pharmacy Technicians-Permissible Duties 4) Subp.4. Written Procedures 5) Subp.5. Supervision	Statute: Vaccine administration is defined in MN Statute 151.01, 27(5) as the practice of pharmacy, and practicing pharmacy without being licensed to do so is a violation of MN Statute 151.34 (13); see also MN Rule 6800.3850. Rule: Vaccine administration is considered the practice of pharmacy, and must be done by a licensed pharmacist per MN Statute 151.01, subds. 3, 15a., and 27(5); see also MN Rule 6800.3850.
**Mississippi**	NEP	PRI	PRI	1) 73-21-73. Definitions 2) 73-21-83 Board to regulate practice of pharmacy; 3) 73-21-111. Personnel 4) Title 30, Part 3001: Pharmacy Practice Regulations, Definitions.1, 54, 59, 61 5) Title 30, Part 3001, Article XXIX, 8: Regulations Governing Institutional Pharmacy-Pharmacy Technicians 6) Article XL: Pharmacy Technicians (1) and (4) Board of Medicine: 7) Miss. Code Ann. §73-43-11 (1972, as amended). 8) Rule 9.2 Position.	N/A
**Missouri**	NEP	PRD	PRD	1) Title XXII Occupations and Professions: 338.010 Practice of pharmacy defined...etc. (1,7,12) 2) 20 CSR 2220-6.050 Administration of Vaccines Per Protocol	N/A
**Montana**	NEP	PRD	PRD	1) 37-7-105. Administration of immunizations 2) 24.174.503 (5) Administration of Vaccines by Pharmacists	N/A
**Nebraska**	NEP	NEP	NEP	1) NRS 38-2891, 38-2837, 38-2866.01 2) 28-013 Pharmaceutical Care Requirements, Title 175, Chapter 8: 8-002 Definitions	N/A
Nevada	NEP	PRD	PRD	1) NRS 639.0113, NRS 639.0124, NRS 639.1371 2) NAC 639.2971 Authorization; contents of and deviation from written protocol.	Yes --> NRS 639 No (Rules)
**New Hampshire**	PRI	PRD	PRD	1) 318:16-b Pharmacist Administration of Vaccines, 2) 318:16-d Pharmacist Administration of Additional Vaccines. 3) Part Ph 1303 Pharmacist Administration of Vaccines Qualifications and Application (c)	Yes --> 318:16-b Yes --> Refer to statute
**New Jersey**	PRI	PRI	PRI	1) 45:14-63 Administration of prescription medication directly to patient, immunization. 2) 13:39-11.13 pharmacy technicians, pharmacy interns, and pharmacy externs; required supervision 3) 13:39-4.21 procedures for physician ordered or government sponsored immunizations performed by pharmacists 4) 13:39-4.21a requirements for pharmacists to administer influenza vaccine to patients under 18 years of age	N/A
**New Mexico**	NEP	NEP	NEP	1) 61-11-11.1. Pharmacy technician; qualifications; duties. (Repealed effective July 1, 2024.) 2) 16.19.22.11 improper activities of pharmacy technicians	N/A
**New York**	PRI	PRI	PRI	1) §6801. Definition of practice of pharmacy, 6803. Practice of pharmacy and use of title "pharmacist". 2) §63.9 Immunizations and emergency treatment of anaphylaxis pursuant to patient specific and non-patient specific orders and protocols.	N/A
**North Carolina**	PRI	PRI	PRI	1) § 90-85.3. Definitions (i1), 2) § 90-85.3A. Practice of pharmacy. (c) 3) § 90-85.15B. Immunizing pharmacists. 4) 21 NCAC 46.2507 administration of vaccines by pharmacists	Yes --> NCGS 90-85.15B only authorizes pharmacists to administer vaccines. There is no grant of authority for pharmacy technicians to do so. No (Rules)
**North Dakota**	PRD	PRI	PRD	1) 43-15-31.5. Injection of drugs - Rules, 43-15-01. Definitions (23, 24) 2) Chapter 61-04-11 Administration of medications and immunizations 3) 61-04-11-01. Definitions (1) 4) 61-04-11-02. 5) 61-02-07.1-06. Tasks pharmacy technicians may not perform 6) 61-02-07.1-05. Tasks pharmacy technicians may perform.	Yes --> NDCC 43-15-31.5 No (Rules)
Ohio	PRD	PRI	PRD	1) ORC 4729.41 Adult immunizations. (1, 2, 3 D.b) 2) 4729-5-38 Immunization and vaccine administration. 3) OAC 4729-5-38 Immunization and vaccine administration.	Yes --> Ohio Administrative Code 4729-5-38 Yes --> Same response for Rules
Oklahoma	NEP	PRI	PRI	1) §59-353.30. Use of agreements - Training requirements and administration of immunizations and therapeutic injections. 2) 535:10-9-13. Administer 3) 535:10-11 (1-6) Pharmacist Administration of Immunizations 4) 535:10-11-4. Immunization registration (a,d)	N/A
**Oregon**	NEP	PRI	PRI	1) 689.005 Definitions. (1), (31) 2) 689.645 Vaccines, patient care services, drugs and devices; formulary; rules 3) 689.655 Power to administer drugs and devices; rules. 4) 855-019-0200: General Responsibilities of a Pharmacist 5) 855-019-0270: Qualifications 6) 855-025-0040 Certified Oregon Pharmacy Technician and Pharmacy Technician Tasks and Guidelines	Yes --> ORS 689.005 ORS 689.155 ORS 689.645 ORS 689.655 Yes --> OAR 855-019-0200 OAR 855-019-0270
**Pennsylvania**	PRD	PRD	PRD	1) Section 9.2. Authority to Administer Injectable (b) 2) 27.403. Conditions for administration. (b)	N/A
**Rhode Island**	NEP	PERM	PERM	1) § 5-19.1-31. Administration of influenza immunizations to individuals between the ages of nine (9) years and eighteen (18) years, inclusive, 2) § 5-19.1-2. Definitions. (w, x) 3) 216-RICR-40-15-1.11 Administration of Immunizations and Performance of Limited-Function Tests by Pharmacists	No No
**South Carolina**	PRD	NEP	PRD	1) 40-43-190 (B,3) Protocol for pharmacists to administer vaccines without order of practitioner; informed consent; records. 2) Board of Pharmacy Approved Technician Duties Policy and Procedure #140 Protocol for administration of vaccines by pharmacists (South Carolina Board of Medical Examiners)-language directly prohibits	N/A
**South Dakota**	NEP	PRD	PRD	1) 36-11-2.2. Practice of pharmacy defined. 2) 36-11-11. Promulgation of rules 3) 36-11-19.1. Authority of registered pharmacists 4) 20:51:28:01. Authority to administer influenza immunizations. 5) 20:51:29:20. Delegation and supervision of technical functions 6) 20:51:29:21. Technical functions 7) 20:51:29:22. Tasks a pharmacy technician may not perform	N/A
**Tennessee**	NEP	NEP	NEP	1) 63-10-204. Definitions 2) 1140-02-.01 pharmacists and pharmacy interns 3) 1140-02-.02 pharmacy technicians	N/A
**Texas**	PRD	PRD	PRD	1) Title 3, Subtitle J. Sec. A554.004. Administration of medication 2) Rule 295.15 Administration of Immunizations or Vaccinations by a Pharmacist under Written Protocol of Physician	Yes --> Sec. 554.004. Administration of medication Yes --> 295.1 Administration of Immunizations or Vaccinations by a Pharmacist under Written Protocol of Physician
**Utah**	NEP	NEP	NEP	1) 58-17b-102. Definitions 2) R156-17b-621. Operating Standards-Pharmacist Administration-Training.	N/A
**Vermont**	NEP	PRI	PRI	1) § 2042b. Pharmacy technicians; nondiscretionary tasks; supervision 2) 10.35 Immunizations 2) 19.6 Coordinating Pharmacist Duties 3) 5.5 “Pharmacy Technician”	No Yes --> Administrative Rules of the Board of Pharmacy 10.35
**Virginia**	PRI	NEP	PRI	1) § 54.1-3320. Acts restricted to pharmacists 2) § 54.1-3321. Registration of pharmacy technicians 3) § 54.1-3300. Definitions 4) 54.1.3401 (Drug Control Act) 5) 18VAC90-21-50 (10) Requirements for Protocols for Administration of Adult Immunizations. 6) 18VAC110-20-111. Pharmacy technicians	Yes --> The Drug Control Act is seen as a permissive act and pharmacy technicians are not authorized to administer vaccines. No --> Rule
Washington	NEP	NEP	NEP	1) RCW 18.64.011 (28) 2) RCW 18.64A.010 3) RCW 18.64A.030 4) WAC 246-901-020 Pharmacy ancillary personnel utilization. 5) WAC 246-863-095 Pharmacist’s professional responsibilities. 6) WAC 246-901-100 Board approval of pharmacies utilizing pharmacy ancillary personnel and specialized functions 7) WAC 246-901-035 Pharmacy technician specialized functions.	Yes --> RCW 18.64 and 18.64A. Yes --> WAC 266-901-020
**West Virginia**	PRD	PRI	PRD	1) 30-5-7. Rule-making authority 2) 15-12-3 Immunizations 3) 15-12-4 Qualifications	N/A
**Wisconsin**	PRD	PRD	PRD	1) 450.035 (2m) Administration of drug products and devices; vaccines. 2) Phar 7.015 Pharmacy technicians.	N/A
**Wyoming**	NEP	PRI	PRI	1) § 33-24-157. Immunization administration. 2) WPA Rules Ch 16 Immunization Regulations, Sec 7 Qualifications	N/A
Washington DC	PRI	PRD	PRD	1) § 3–1201.02. Definitions of health occupations. 2) § 3–1202.08. Board of Pharmacy and Advisory Committee on Clinical Laboratory Practitioners. 3) Chapter 99: Pharmacy Technicians-9910.3-Scope of Practice. 4) Chapter 65: Pharmacists-6512-Administration of immunizations and vaccinations by pharmacists	Yes --> District of Columbia Municipal Regulations 9910.3 (g) Yes --> same as above

Note: Permissive = PER, Prohibited Directly = PRD, Prohibited Indirectly = PRI, Not Expressly Prohibited = NEP.
